# The Self-Perception and Usage of Medical Apps amongst Medical Students in the United States: A Cross-Sectional Survey

**DOI:** 10.1155/2016/3929741

**Published:** 2016-09-05

**Authors:** Cara Quant, Lisa Altieri, Juan Torres, Noah Craft

**Affiliations:** ^1^Department of Medicine, Cedars Sinai Medical Center, Los Angeles, CA, USA; ^2^Kaiser Permanente, Los Angeles Medical Center, Los Angeles, CA, USA; ^3^Charles R. Drew University of Medicine and Science, Los Angeles, CA, USA; ^4^Los Angeles Biomedical Research Institute at Harbor-UCLA Medical Center, Los Angeles, CA, USA

## Abstract

*Background*. Mobile medical software applications (apps) are used for clinical decision-making at the point of care.* Objectives*. To determine (1) the usage, reliability, and popularity of mobile medical apps and (2) medical students' perceptions of app usage effect on the quality of patient-provider interaction in healthcare settings.* Methods*. An anonymous web-based survey was distributed to medical students. Frequency of use, type of app used, and perceptions of reliability were assessed via univariate analysis.* Results*. Seven hundred thirty-one medical students responded, equating to a response rate of 29%. The majority (90%) of participants thought that medical apps enhance clinical knowledge, and 61% said that medical apps are as reliable as textbooks. While students thought that medical apps save time, improve the care of their patients, and improve diagnostic accuracy, 53% of participants believed that mobile device use in front of colleagues and patients makes one appear less competent.* Conclusion*. While medical students believe in the utility and reliability of medical apps, they were hesitant to use them out of fear of appearing less engaged. Higher levels of training correlated with a greater degree of comfort when using medical apps in front of patients.

## 1. Introduction

Medical applications (apps) have been suggested to support clinical decision-making, improve communication between hospital staff, and improve patient management [1–3]. Studies have indicated frequent use of apps as reference and information management tools in clinical practice, with a trend toward increasing app use amongst medical practitioners with less training [4–6]. Resident physicians have reported increasing usage of apps in their clinical practice, most commonly drug guides, medical calculators, coding and billing apps, and pregnancy wheels [6]. While previous literature has examined the utility of apps for physicians in various medical specialties, less is known about the popularity, reliability, and usage of medical apps by medical students [1, 2, 6–8].

While the majority of medical students own smartphones, use medical apps at least once a day, and agree that having a smartphone has a positive effect on their education, it has been suggested that medical students have conflicting negative perceptions on smartphone utilization in the hospital setting [9, 10]. One of the most common recurring negative theme that surfaced in a survey of medical students in the United Kingdom was the fear of appearing disinterested in patient care while using mobile apps [10, 11].

The apparent lack of insight into medical students' opinions regarding medical applications is surprising, given the popularity of smartphone usage in the medical setting. This study aimed to determine the reliability and usage of medical apps in a large, nationwide cohort of United States medical students. Specifically, the study aimed to report (1) the usage, reliability, and popularity of mobile medical apps and (2) medical students' perceptions on app usage and its effect on the quality of patient-provider interaction.

## 2. Materials and Methods

This cross-sectional study used an online survey that was administered to medical students in United States accredited MD or DO programs from December 2014 to January 2015. The survey was offered to approximately 2500 subjects who were contacted through medical student organizations. Of the 2500 students contacted, 731 (29%) responded. The survey, shown in Table 1, was approved by the Charles R. Drew University of Medicine and Science, Institutional Review Board. The survey was in English only; therefore subjects were English speaking. There were no inclusion or exclusion criteria based on specialty, race/ethnicity, or gender.

Consenting subjects were emailed an 18-question online survey (Table 1), using a commercial, web-based, surveying instrument called SurveyMonkey. All data was collected during a 2-month time period and subjects were given two reminders to complete the survey. Subjects were incentivized with the chance to win a portable electronic device.

The survey was constructed by the principal investigators and was reviewed by an expert panel for content validity and reliability. Questions were derived from previous literature [10, 12], and the eleven apps named in the survey were derived from a list of the most popular medical apps on the iTunes store, imedicalapps.com, and other websites [13, 14]. Questions were multiple choice, and some utilized a Likert scale format. Prior to administration, the survey was piloted within one hospital and altered accordingly.

After data collection was complete, univariate analysis was performed and all numerical data were reported as simple statistical data (e.g., frequency, percentage, and standard deviation).

## 3. Results

Over the two-month period, a total of 731 responses were received, with a response rate of 29% (731/2500). Equal numbers of participants were preclinical students (1st-2nd year of school) and clinical students (3rd-4th year of school). The majority (58%) were women, and most (89%) were under 30 years old.

### 3.1. Usage and Reliability

Ninety percent (660/731) of participants agreed or strongly agreed that medical apps enhance clinical knowledge. A minority of participants (23%) believed that medical apps are more reliable than textbooks, and 61% believed that apps are as reliable as textbooks. Amongst the eleven apps assessed in the survey, UpToDate (78%) and Epocrates (61%) were the most widely used medical apps. The five apps perceived to be the most trustworthy were UpToDate (97%), Epocrates (93%), DynaMed (83%), Micromedex (82%), and Medscape (81%) (Table 2). Smaller percentages of respondents considered Wikipedia, WebMD, and Google to be trustworthy (41%, 33%, and 29%, resp.).

### 3.2. Medical App Use with Colleagues

Ninety-five percent of medical students who responded said medical app use saves time, 87% reported that it improves patient care, and 78% believed it increases diagnostic accuracy (Table 3). The general clinic setting referred to in this study is the clinical environment outside direct patient-physician interaction (e.g., discourse between colleagues and acquisition of medical information). More than 50% of participants believed their use of a mobile device made them appear less engaged in front of colleagues.

### 3.3. Medical App Use with Patients

The same percentage (54%) of both groups of medical students reported that using medical apps in front of patients makes the user appear less engaged. Interestingly, 35% of first- and second-year students reported feeling comfortable using medical apps in front of a patient, while 56% of third and fourth year students reported feeling comfortable. More experienced medical students (i.e., third and fourth years) thought that patients had more favorable opinions of app use, compared to less experienced medical students (i.e., first and second years). While 36% of preclinical students thought that app use in front of patients made them appear “fresh out of training,” only 55% of clinical students reported this statement. More experienced students reported that app use in front of patients shows that a clinician “cares enough to double check,” whereas only 36% of less experienced students agreed with this statement. Approximately equal percentages of preclinical and clinical students reported that app use makes a clinician appear tech savvy (42% and 41%, resp.), as well as incompetent (52% and 51%, resp.).

## 4. Discussion

Clinicians are increasingly using medical apps in the clinical setting, yet little is known about medical students' usage and perceptions of medical apps. Our cross-sectional survey of United States medical students found that UpToDate and Epocrates are the most commonly used and trusted apps amongst medical students. The majority of participants thought that medical apps save time, improve care of their patients, and improve diagnostic accuracy. However, participants also felt that mobile device use in the clinical setting makes them appear less engaged.

Medical students with more experience (i.e., third and fourth year students) were more comfortable using apps during patient encounters, compared to first- and second-year students which is consistent with previous research. This may be because less experienced students are more aware of their gaps in knowledge and thus may be more concerned with patients' perceptions of their competency.

The point-of-care access to medical information has led to improved decision-making, enhanced telemedicine capabilities, and decreased hospital stays [15]. If health systems want to reap these rewards associated with app use, the culture of medicine must embrace technology. Some medical schools and residency programs have encouraged app use by requiring all medical students to have certain apps on their phones and often provide free mobile subscriptions to these apps [16]. While cultural attitudes toward technology may be changing, it is possible that mobile technology has not been fully integrated into the culture of medicine. As the US healthcare system evolves and places a priority on evidence-based medicine and technology, it becomes increasingly important to have baseline data on medical app use in the country's next generation of physicians. Some institutions are already looking into ways to integrate new learning modalities to teach medical students who grew up in the generation deemed “Net Generation” about the efficacy of using medical apps to supplement learning. One such study done by Briz-Ponce et al. [17] found improved test scores in those who utilized mobile apps versus the traditional structure of teaching. Another study by Gutmann et al. [18] on the use of digital versus nondigital learning resources amongst medical students found that there is a high prevalence of digital learning resources such as online lecture slides, e-learning cases, and mobile app use that are favored over textbooks. Although more studies are needed to better understand the impact of mobile technology in medical education and as a tool for medical professionals, there seems to be a growing demand to integrate the two. Specifically, this data will allow us to develop and recommend appropriate learning materials and activities for delivery on smartphone applications.

## 5. Limitations

Given that the survey was administered via email with an incentive offered upon completion, there was a voluntary response bias of participants. Without access to a list of all US medical schools' offices, the schools surveyed were selected through a convenience sample, which suggests that the population is unlikely to be representative of all medical students. Due to sampling bias, the sample may not be representative of the entire population of US medical students and results in low external validity of the study. Because the survey was conducted through a web-based medium, it was likely to be filled out by technologically inclined medical students. The response rate was relatively low but the number of responses received is comparable to similar surveys of this kind [19]. Despite these limitations, the data obtained from our convenience sample provides baseline data for future research.

## 6. Conclusion

Point-of-care apps have been shown to augment patient care, save time, and increase diagnostic accuracy. While medical students regard medical apps favorably, they also hesitate to use apps during patient encounters and with peers, due to a fear of being perceived as incompetent. Considering that medical apps have been shown to increase diagnostic accuracy, there is a need for medical schools and hospitals to integrate apps into medical education.

## Figures and Tables

**Table 1:** Survey questions.

Question	Answer choices
(1) What resources do you use to look up medical information? (Check all that apply)	Textbooks, Journal Articles, Medical Apps, Websites

(2) What medical apps do you use on a regular basis? (Check all that apply)	Epocrates, UpToDate, VisualDX, Dynamed, Diagnosaurus, Lexicomp, Pepid, Eponymns, Isabel, Medscape, Micromedex, None

(3) Rate these medical apps/websites for how much you trust the information (1–5 do not trust–completely trust)	Epocrates, UpToDate, VisualDX, Dynamed, Diagnosaurus, Lexicomp, Eponymns, Medscape, WebMD, Micromedex, Wikipedia, Google

(4) How much time does your medical school/residency training program formally devote to teaching the appropriate use of information technology in medical decision making?	0 hours, 1–5 hours, >5 hours

(5) Using medical apps enhances your knowledge as a medical student/resident	Strongly Agree, Agree, Neither Agree nor Disagree, Disagree, Strongly Disagree

(6) How would you compare the information obtained from medical apps with information obtained from a textbook?	More Reliable, Same Reliability, Less Reliable

(7) Are you comfortable using medical apps during a patient encounter?	Very Comfortable, Neither Comfortable Nor Uncomfortable, Uncomfortable, Very Uncomfortable

(8) How often do you use a mobile device (smartphone, tablet, etc.) to (a) look up information; (b) share information	Not at all, 1-2x per week, daily, multiple times per day

(9) Usage of medical apps can (a) save you time; (b) improve the care of my patients; (c) increase diagnostic accuracy	Strongly Agree, Agree, Neither Agree nor Disagree, Disagree, Strongly Disagree

(10) What do patients think of you when using a medical app in front of them? (Check all that apply)	You are tech savvy and modern, You don't know what you are doing, You care enough to double check you work, You are fresh out of training

(11) Using a medical app on a mobile device (smartphone, tablet, etc.) in front of a colleague makes you appear less engaged	Strongly Agree, Agree, Neither Agree nor Disagree, Disagree, Strongly Disagree

(12) Older doctors have a harder time adapting to new technologies and still rely on their memory for most medical decisions.	Strongly Agree, Agree, Neither Agree nor Disagree, Disagree, Strongly Disagree

(13) Younger doctors are more comfortable using mobile technologies to access knowledge	Strongly Agree, Agree, Neither Agree nor Disagree, Disagree, Strongly Disagree

(14) Where do you look for help in creating a differential diagnosis? (Check all that apply)	UpToDate, VisualDX, Dynamed, Diagnosaurus, eMedicine Isabel, Medscape, None

**Table 2:** Perceptions of medical app reliability amongst US medical students (*N* = 731).

Application name	Do not trust% (total)	Indifferent% (total)	Trust% (total)	Total
Epocrates	1 (4)	6 (35)	93 (519)	558
UpToDate	0 (2)	3 (16)	97 (634)	652
VisualDx	0 (0)	20 (42)	80 (166)	208
DynaMed	0 (1)	17 (38)	83 (187)	226
Diagnosaurus	2 (3)	38 (56)	60 (87)	146
Lexicomp	0 (0)	25 (38)	75 (113)	151
Eponymns	2 (2)	46 (45)	53 (52)	99
Medscape	2 (12)	16 (89)	82 (457)	558
Micromedex	0 (0)	18 (35)	82 (162)	197
WebMD	27 (169)	40 (256)	33 (212)	637
Wikipedia	20 (140)	38 (268)	42 (291)	699
Google	17 (117)	54 (360)	29 (195)	672

**Table 3:** Perceptions of medical app use amongst US medical students (*N* = 731).

Answers	Disagree% (total)	Indifferent% (total)	Agree% (total)	Total
Save you time	1 (11)	4 (33)	95 (765)	809
Improve the care of my patients	2 (12)	11 (90)	87 (707)	809
Increase diagnostic accuracy	2 (15)	20 (160)	78 (632)	807
